# Real-time PCR for diagnosis of imported schistosomiasis

**DOI:** 10.1371/journal.pntd.0007711

**Published:** 2019-09-11

**Authors:** Hélène Guegan, Judith Fillaux, Eléna Charpentier, Florence Robert-Gangneux, Pamela Chauvin, Emilie Guemas, Jérôme Boissier, Alexis Valentin, Sophie Cassaing, Jean-Pierre Gangneux, Antoine Berry, Xavier Iriart

**Affiliations:** 1 Univ Rennes, CHU Rennes, Inserm, EHESP, IRSET (Institut de Recherche en Santé Environnement et Travail) – UMR_S 1085, Rennes, France; 2 Service de Parasitologie-Mycologie, CHU Toulouse, Toulouse, France; 3 Centre de Physiopathologie de Toulouse Purpan (CPTP), Université de Toulouse, CNRS, INSERM, UPS, Toulouse, France; 4 Université de Perpignan Via Domitia, IHPE UMR 5244, CNRS, IFREMER, Université de Montpellier, Perpignan, France; University of Nottingham, UNITED KINGDOM

## Abstract

**Background:**

The diagnosis of schistosomiasis currently relies on microscopic detection of schistosome eggs in stool or urine samples and serological assays. The poor sensitivity of standard microscopic procedures performed in routine laboratories, makes molecular detection methods of increasing interest. The aim of the study was to evaluate two in-house real-time *Schistosoma* PCRs, targeting respectively *S*. *mansoni* [Sm] and S. *haematobium* [Sh] in excreta, biopsies and sera as potential tools to diagnose active infections and to monitor treatment efficacy.

**Methods:**

*Schistosoma* PCRs were performed on 412 samples (124 urine, 86 stools, 8 biopsies, 194 sera) from patients with suspected schistosomiasis, before anti-parasitic treatment. Results were compared to microscopic examination and serological assays (enzyme-linked immunosorbent assay (ELISA), indirect haemagglutination (HA) and Western Blot (WB) assay).

**Results:**

Compared to microscopy, PCRs significantly increased the sensitivity of diagnosis, from 4% to 10.5% and from 33.7% to 48.8%, for Sh in urine and Sm in stools, respectively. The overall sensitivity of PCR on serum samples was 72.7% and reached 94.1% in patients with positive excreta (microscopy). The specificity of serum PCR was 98.9%. After treatment, serum PCR positivity rates slowly declined from 93.8% at day 30 to 8.3% at day 360, whereas antibody detection remained positive after 1 year.

**Conclusion:**

*Schistosoma* PCRs clearly outperform standard microscopy on stools and urine and could be part of reference methods combined with WB-based serology, which remains a gold standard for initial diagnosis. When serological assays are positive and microscopy is negative, serum PCRs provide species information to guide further clinical exploration. Biomarkers such as DNA and antibodies are of limited relevance for early treatment monitoring but serum PCR could be useful when performed at least 1 year after treatment to help confirm a cured infection.

## Author summary

Schistosomiasis is one of the most important human parasitic neglected tropical diseases. It is a major source of morbidity and mortality in Africa but also in South America, the Caribbean, the Middle East, and Asia. It is transmitted by skin penetration of schistosome cercariae via contact with freshwater. *Schistosoma mansoni* and *S*. *haematobium* are the most common species and are frequent causes of infection in travelers and migrants returning from endemic areas. Chronic infections with these two species can cause irreversible damage to the liver or genitourinary tract. Diagnosis mainly relies on serological screening and microscopic procedures from urine and stool specimens that can, however, fail to detect low parasite burden and depend on operator competence. So there is a need to improve the detection of this disease. With this retrospective study, we evaluate the accuracy of a specific *Schistosoma* PCR assay for the diagnosis of schistosomiasis on a large cohort of migrants and travelers returning from endemic areas. Our study showed that PCR, a technique allowing *Schistosoma* DNA amplification and detection, greatly improved the diagnosis of both parasite species in urine, feces and biopsies. We also demonstrate that the detection of circulating *Schistosoma* DNA in blood by PCR is useful to confirm schistosomiasis diagnosis, to provide a species identification when the microscopy research is negative and to monitor the treatment efficacy.

## Introduction

Schistosomiasis is a snail-borne parasitic disease of great concern worldwide, occurring mainly in Africa, Asia, and to a lesser degree in South America and the Middle East [1,2]. In non-endemic areas, there is a growing number of people potentially infected, given the increasing number of immigrants, foreign workers, and travelers. Recently, the emergence of urogenital schistosomiasis, due to *S*. *haematobium and S*. *bovis* hybrids, has been reported in a previously uninfected area in Corsica (France), with more than 120 confirmed cases among local people and tourists [3,4].

The diagnosis of urogenital and intestinal schistosomiasis currently relies on microscopy procedures which are time-consuming, require trained operators and offer poor sensitivity, particularly when the parasite burden is low. Additionally, egg release and migration from tissues to the lumen (gut, bladder) is a long process (>6 weeks) [2], leading to diagnosis delay. The detection of specific antibodies is the most commonly applied alternative diagnostic approach in non-endemic routine laboratories [5,6]. Among the current panel of serological techniques which are highly sensitive, the development of an immunoblotting assay using *S*. *mansoni* and *S*. *haematobium* adult worm extracts improved the performances of serological screening and proved to be relevant in both urogenital and intestinal schistosomiasis [7,8]. Although the usefulness of serological tools has been demonstrated, particularly for symptomatic travelers, their contribution to diagnosing patients living in endemic regions is limited due to their inability to differentiate between ongoing and previous infections. Moreover, in endemic populations, circulating adult worm-derived antigens have been reported to be good indicators of active infection, but still appear to lack sensitivity, especially in case of low parasite burden [9–11]. Excellent results were described in diagnosing *S*. *mansoni* infections by the detection of circulating cathodic antigens in urine and serum samples [9], but the performance was disappointing in *S*. *haematobium* detection [12].

The development of molecular methods to improve the diagnosis of parasitic diseases has been encouraging active research in recent years [13,14]. The detection of *Schistosoma* DNA by PCR in stools and urine appears to be a promising, highly sensitive, and specific tool that could improve and facilitate the diagnosis of schistosomiasis, particularly in non-endemic countries where the parasite burden can be lower [15–18]. A few previous studies demonstrated the potential interest of blood-based PCR for the diagnosis of acute schistosomiasis. As evidenced by Wichmann *et al*., the high potential of both *Schistosoma haematobium* (Sh) and *Schistosoma mansoni* (Sm) PCR assays is linked to the high repetition of the multicopy targeted genes, accounting for 12–15% of the whole *Schistosoma* genome [19,20].

This work aimed i) to assess the performance of *Schistosoma* PCR assays for the diagnosis of imported schistosomiasis in comparison to conventional microscopy in stool, urine and biopsy samples, ii) to evaluate the clinical relevance of *Schistosoma* DNA detection in the serum for the diagnosis of active infection and for the monitoring of treatment efficacy.

## Materials and methods

### Ethics statement

The study was approved by the ethics committee of the Rennes University Hospital (No. 18.111). Biological material was obtained only for standard diagnosis on the basis of physicians’ prescriptions. Clinical data were made anonymous for analysis. According to French Public Health Law [21], protocols of this type are exempt from the requirement for formal informed consent.

### Study design

Data from patients with suspected schistosomiasis examined at Toulouse and Rennes University Hospitals between January 2016 and March 2018 were collected. Only patients with at least a *Schistosoma* PCR and a serology analysis (including Western Blot), alone or combined with excreta examination, and without any previous anti-*Schistosoma* treatment were retained in the study. The diagnostic procedures were part of the standard diagnostic work-up for schistosomiasis investigation as described below.

The design of this study aimed to evaluate the performances of *Schistosoma* detection by PCR in two contexts: i) in stool, urine and biopsy samples in comparison to conventional microscopy diagnosis, and ii) in the serum for the diagnosis of active infection and monitoring of treatment efficacy.

For the evaluation of PCRs assays in sera, patients were classified according to results of microscopy, serology and blood eosinophil count. “Proven active *Schistosoma* infection” was defined by the microscopic detection of *Schistosoma* eggs in excreta or biopsy samples. “Probable active infection” was defined by a positive *Schistosoma* WB serology associated with a blood eosinophil count ≥0.5G/L, while cases were graded as “strictly serological schistosomiasis” when the WB was positive without blood eosinophil increase. Patients with “excluded schistosomiasis” were defined by a negative *Schistosoma* WB serology or both negative ELISA and HA, and negative microscopic egg detection in excreta, when performed.

In addition, an evaluation of PCR specificity was conducted in serum, stool and urine samples recovered from volunteers who had never visited schistosomiasis-endemic areas.

All the diagnostic tests (microscopy, detection of anti-*Schistosoma* antibodies including WB and *Schistosoma* PCR assays) were performed independently and blindly from one another on each anonymised sample. The same samples were used (excreta, biopsies, sera) for *Schistosoma* PCR assays or reference methods (microscopy, detection of anti-*Schistosoma* antibodies including WB).

### Microscopy (ME)

Microscopic examination for the presence of *Schistosoma* eggs in urine samples was performed by examination of the pellet from micturition obtained after centrifugation. Fresh stool specimens were examined after 2 concentration techniques (flotation and diphasic methods) [22]. Direct examination of bladder, gut and other biopsies was performed after mechanical grinding. Results of histological examination after biopsy staining were also collected, when performed.

### Detection of anti-*Schistosoma* antibodies

Sera were screened for anti-*Schistosoma* antibodies using the *Schistosoma* IgG ELISA (Bordier Affinity Products, Crissier, Switzerland *or Schistosoma* Antibody Detection Test Kit, Scimedx, Dover, New Jersey) and the indirect hemagglutination (HA) test (Schistosomiasis Fumouze, Fumouze Diagnostics, Levallois-Perret, France) according to manufacturers’ instructions. Sera with a positive or a doubtful result by at least one technique were then confirmed using a Western Blot (WB) assay (Schisto II Western Blot IgG kit (LD-BIO Diagnostics, Lyon France)), according to the manufacturer’s instructions. In total, the serology was considered positive if the WB was positive. A serum was considered negative if the two screening techniques or the WB were negative.

### DNA extraction

Two hundred fifty to 500 mg of stool sample were suspended in 1 mL of PBS and frozen at -20°C before DNA extraction. Extraction was performed on 200 μL of stool suspension after bead-beating, using High Pure PCR Template Preparation Kit (Roche Diagnostics, Meylan, France) according to the manufacturer’s instructions.

For blood and urine, DNA extraction was performed using 1 mL of serum or 1 mL of urine pellet and eluted in a volume of 50 μL, using the MagNA Pure Compact Nucleic Acid Isolation Kit (Roche Diagnostics) on a MagNA Pure Compact Instrument (Roche Diagnostics).

### Extraction/Inhibition control

DNA extracts were first screened for PCR inhibitors using the beta-globin gene amplification, as previously described [23]. Briefly, the run was performed in a 10 μL reaction mix containing 2.5 μL DNA, 10X Taq SyBr, 0.1 μM of BgO7 and BgO8 primers and 4 mM of MgCl_2_. Amplification was conducted on a LightCycler 2 instrument (Roche Diagnostics) during 40 cycles of 15 s at 95°C, 10 s at 70°C and 20 s at 72°C, followed by a fusion step.

As long as the beta-globin gene was detected, DNA extracts were considered suitable for *Schistosoma* PCR testing. In case of PCR inhibition (lack of amplification of beta-globin gene), samples were retested diluted to 1:4, and the result was considered uninterpretable if beta-globin remained undetected.

### *Schistosoma* real-time PCR assays

Two species-specific in-house real-time PCRs were evaluated. S. *mansoni* DNA detection (SmPCR) was performed using primers (SRA1 and SRS2) and probe (SRP) from Wichmann *et al*., targeting the Sm1-7 tandem repeat sequence [24]. The repetitive *Dra1* sequence of S. *haematobium* (ShPCR) was amplified using primers (Sh-FW and Sh-RV) from Hamburger *et al*. [20], and probe from Cnops *et al*. [25].

PCR assays were performed in a 20 μL reaction mix containing 5 μL DNA, 10X LightCycler FastStart DNA Master HybProbe master mix, 5 mM of MgCl_2_, 0.5 μM of each primer and 0.25 μM of probe. Amplification was conducted on a LightCycler 2 instrument (Roche Diagnostics) and consisted of 10 min at 95°C and 45 cycles of 15 s at 95°C and 1 min at 60°C. A test was positive when the threshold was attained within 45 PCR cycles (Ct-value<45).

### Statistical analysis

Data were analyzed using GraphPad Prism software. Data were expressed as median with interquartile range [25^th^;75^th^ percentile]. Nonparametric Mann-Whitney and Kruskal-Wallis tests were performed for distribution comparison. For proportion comparisons, the chi-square test or Fisher’s exact test was used, as appropriate. Kappa coefficient was used to measure the agreement between each test. Correlation between blood eosinophil count and PCR Ct values was assessed with the Spearman rank correlation test. Differences were considered statistically significant if the p-value was < 0.05. DNA burden during post-treatment follow-up was calculated as the ratio of (45-Ct value after treatment)/(45-Ct value before treatment)*100.

## Results

### Patients

A total of 412 clinical samples (urine, stool, biopsy and serum samples) from 255 patients were analyzed for schistosomiasis diagnosis.

**Fig 1:**
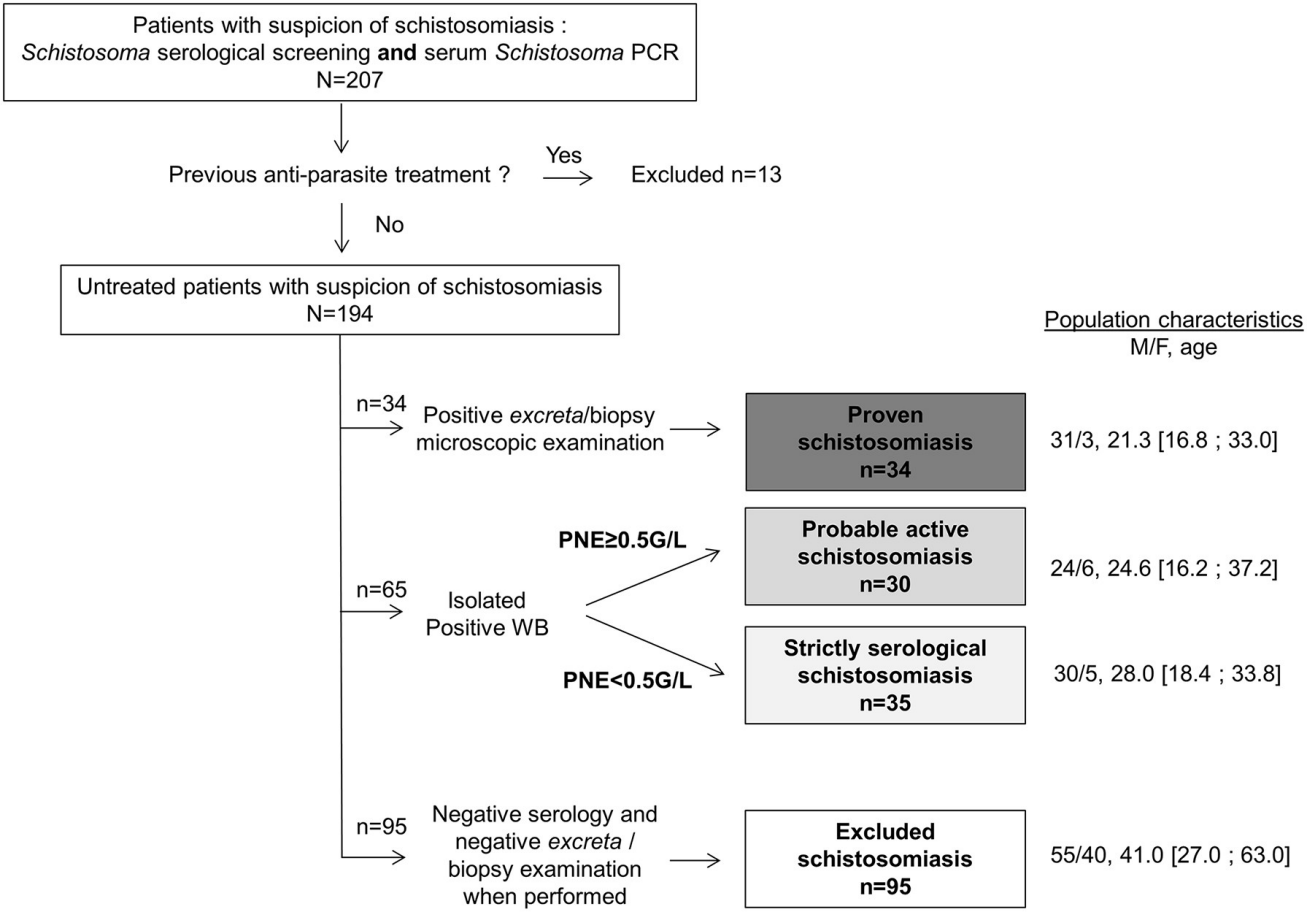
Flow chart for serum PCR evaluation. PNE: polynuclear eosinophil blood count.

The study population for PCR evaluation in excreta consisted of 180 patients (Table 1). The performance of serum PCR was assessed on a series of 194 sera from 194 patients, graded as “proven active schistosomiasis” (n = 34), “probable active schistosomiasis” (n = 30), “strictly serological schistosomiasis” (n = 35) or “excluded schistosomiasis” (n = 95), according to serology, blood eosinophil count and *Schistosoma* detection in excreta by microscopy (Fig 1). Of all 99 suspected cases, 82 were immigrants, mainly young men (Fig 1), 11 patients were travelers for a period of ≤3 months, 3 were French expatriates for more than 7 years, and the length of stay was unknown for the 3 remaining patients. As for the visited endemic areas, most patients visited Africa (n = 92; 93%); others visited Corsica (n = 3; 3%) and other areas (n = 4; 4%).

**Table 1:** Performance of PCR and microscopic examination for the detection of *S*. *mansoni* and *S*. *haematobium* in urine, stool and biopsy samples.

Sample (N)	Patients (n)	Positive microscopy, n/N (%)	Positive SmPCR or ShPCR n/N (%)	Agreement PCR/microscopy (%)
Urine (N = 124)	108	5^a^/124 (4.0)	13^b^ /124 (10.5)	93.5
Stool (N = 86)	65	29^c^/86 (33.7)	42^d^/86 (48.8)	84.9
Biopsy (N = 8^e^)	7	2^f^/8 (25.0)	2/8 (25.0)	100

SmPCR: *S*. *mansoni* PCR; ShPCR: *S*. *haematobium* PCR

^a^5/5 positive-microscopy samples yielded positive PCR

^b^Two samples were simultaneously positive for SmPCR and ShPCR; p<0.001 compared to microscopy

^c^29/29 positive-microscopy samples yielded positive PCR

^d^p<0.001 compared to microscopy

^e^Rectum (n = 3); colon (n = 2); rectum/colon (n = 1); perineal abscess (n = 1) and liver (n = 1)

^f^Rectum (n = 1) colon (n = 1) collected from the same patient. 2/2 positive-microscopy samples yielded positive PCR

### Evaluation of PCR specificity

The specificity of both Sm and ShPCR assays was evaluated on 3 biological matrices, i.e. serum, urine and stool samples, from subjects unlikely to have had previous contact with the parasite. The specificity was 100%, as neither of the two PCR assays yielded a positive result in the 20 serum, 20 urine, and 20 stool samples. Additionally, PCR specificity was also evaluated regarding other parasites, in 3 blood samples and 34 stools samples in which parasites had been detected by other diagnostic methods. No cross reaction was observed with *Toxoplasma (n = 1)*, *Plasmodium (n = 1)*, *Leishmania (n = 1)*, *Enterocytozoon bieneusi (n = 1)*, *Encephalitozoon sp (n = 1)*, *Cryptosporidium sp (n = 1)*, *Endolimax nana (n = 2)*, *Blastocystis sp (n = 4)*., *Entamoeba hartmanni (n = 1)*, *Entamoeba dispar (n = 1)*, *Entamoeba histolytica (n = 1)*, *Entamoeba coli (n = 1)*, *Dientamoeba fragilis (n = 4)*, *Giardia intestinalis (n = 5)*, *Enterobius vermicularis (n = 1)*, *Ascaris lumbricoides (n = 2)*, *Trichuris trichiura (n = 1)*, *Strongyloides stercoralis (n = 2)*, *Ancylostomidae (n = 1)*, *Hymenolepis nana (n = 3) and Taenia sp*.*(n = 2)*.

### Performances of PCR assays compared to microscopy in urine, stool and biopsy samples

A total of 124 urine, 86 stool and 8 biopsy samples were collected from 108, 65, and 7 patients, respectively, with suspected infection with *Schistosoma*, before any anti-parasite treatment. A comparison of egg detection performance using microscopic methods (microscopic examination or anatomic pathology procedures) and *Schistosoma* DNA detection is presented in Table 1. All PCR results were interpretable.

Of the 124 urine samples, 13 (10.5%) yielded a positive ShPCR result, whereas parasite eggs were microscopically detected in only 5 samples (4%) (p<0.001). Two samples were positive by both PCRs. The 8 samples with discordant results were collected from 7 distinct patients, all having a positive WB-based serology, suggesting that these were 8 true-positive samples detected by PCR but not by microscopy. All positive-microscopy samples were positive by PCR.

In stools, 42 out of 86 stool samples (48.8%) yielded a positive SmPCR but only 29 with microscopy (33.7%) (p<0.001) (Table 1). For the 13 samples with discordant results, WB-based serology supported positive PCR results. All samples containing *S*. *mansoni* eggs yielded a positive SmPCR result. No positive ShPCR was found in stool samples.

The concordance of SmPCR and microscopy for biopsies was 100%, as the 2 colon and rectal specimens, sampled from the same patient, were positive both by direct examination and SmPCR.

The agreement between the results of microscopy and PCR assays varied between 84.9 and 100% according to the type of sample. Discordances concerned only probable true-positive samples detected by PCR but not by microscopy (Table 1). Therefore the specificity of PCR can be considered as 100%.

Moreover, 8 patients gave several stool or urine specimens (2 or 3) a few days apart (Table 2). Interestingly, the PCR consistently yielded a positive signal (except patient 5), whereas eggs were inconsistently detected by microscopy in 7/8 patients.

**Table 2:** Results of microscopic detection and *Schistosoma* PCR in iterative *excreta* samples.

Patient	Matrix	Number of positive samples n/N	
		Microscopy	PCR
1	Urine	1/2	2/2
2	Stool	1/3	3/3
3	Stool	2/3	3/3
4	Stool	1/3	3/3
5	Stool	1/3	2/3
6	Stool	0/3	3/3
7	Stool	0/2	2/2
8	Stool	2/2	2/2

PCR Ct values appeared to be correlated with microscopic results. Indeed, median Ct values of both PCR assays were significantly lower in samples with microscopic egg detection, compared to those with negative microscopic results: 19.5 [17.4; 21.3] *versus* 21.6 [19.8; 26.0] (p = 0.019) for Sm detection in stools, 17.1 [15.7; 17.8] *versus* 21.5 [20.7; 26.8] (p = 0.003) for Sh detection in urine.

Overall, these results suggested that the molecular detection of *Schistosoma* by PCR is more efficient and reliable than microscopic examination of excreta, particularly in the case of low burden infections.

### Performances of serum PCR compared to serology and excreta examination for schistosomiasis diagnosis

The overall rate of positive serum PCRs among the 99 patients with a microscopic and/or serological criterion in favor of schistosomiasis was 72.7% (72/99) (Table 3). All PCR results were interpretable. The specificity was excellent, reaching 98.9%. The only serum with a positive SmPCR among cases with excluded schistosomiasis was unlikely to be a false-positive result, as the ELISA serology was simultaneously positive, and the patient had been living for 28 years in Sudan and had abdominal pain. This patient was classified as “excluded schistosomiasis” because of a negative WB (probably a false-negative result).

**Table 3:** Rate of positive serum PCRs in the various clinical categories, according to *Schistosoma* species (n = 194 patients).

Diagnosis	Number of positive PCR in serum n/N (%)			Median Ct value for both PCRs
	SmPCR and/or ShPCR	ShPCR	SmPCR	
Schistosomiasis (N = 99)	72^a^/99 (72.7)	22/99 (22.2)	56/99 (56.6)	34.1 [32.0; 36.1]
Proven N = 34	32/34 (94.1)	13/34 (38.2)	22/34 (64.7)	32.9 [30.9; 35.1]
Urogenital N = 13	11/13 (84.6)	11/13 (84.6)	1^b^/13 (7.7)	
Intestinal N = 18	18/18 (100.0)	0/18 (0)	18/18 (100)	
Mixed infection N = 2^c^	2/2 (100)	2/2 (100)	2/2 (100)	
Undetermined N = 1^d^	1/1 (100)	0/1 (0)	1/1 (100)	
Probable active N = 30	23^e^/30 (76.7)	5/30 (16.7)	19/30 (63.3)	34.7 [32.3; 36.6]
Strictly serological N = 35	17^f^/35 (48.6)	4/35 (11.4)	15/35 (42.9)	35.0 [32.2; 36.2]
Excluded schistosomiasis N = 95	1/95 (1.1)	0/95 (0)	1/95 (1.1)	NA

NA: not applicable

^a^6/72 patients had simultaneously positive Sm and ShPCR

^b^One serum sample simultaneously yielded positive results for both Sm and ShPCR

^c^Two patients had a concomitant detection of Sm in stool and Sh in urine

^d^One patient had a positive Sm PCR whereas eggs of Sh or *S*. *intercalatum* were microscopically detected in the rectal biopsy

^e^1/30 was positive for Sm and ShPCR

^f^2/35 were positive for Sm and ShPCR

Interestingly, serum PCRs were positive in 85.9% (55/64) of active schistosomiasis (proven and probable groups), exceeding the microscopy yield which was only 53.1% (34/64) (Table 3). In the group of proven schistosomiasis, the positivity rate of serum PCRs reached 94.1% (32/34). Among the 2 missed PCR detections (2/34), the two occurred in patients with proven urogenital schistosomiasis (Sh eggs in urine), including one with a concomitant positive urine ShPCR (the urine ShPCR was not performed for the second one).

In the group of proven schistosomiasis, all patients having a positive microscopy had a positive WB, except in one case. The patient missed by the WB-serology had a positive serum ShPCR, an ELISA index value in grey zone and a positive egg detection in vesical biopsy, associated to urinary symptoms (macroscopic hematuria and micturition disorders for 6 months).

Overall, the sensitivity of serum PCR significantly exceeded the microscopy, leading to a low agreement percentage and kappa coefficient of 77.8% and 0.472 respectively (Table 4). Indeed, in patients with negative microscopy, the serum PCR positivity rate reached 76.7% (23/30) and 48.6% (17/35), respectively in “probable” and “strictly serological schistosomiasis” groups (Table 3). However, the level of circulating *Schistosoma* DNA was not significantly higher in patients with positive egg detection (proven cases) and/or with increased blood eosinophil count, as the median Ct values did not differ significantly between groups: 32.9 [30.9; 35.1] (proven cases), 34.7 [32.3; 36.6] (probably active cases), 35.0 [32.2; 36.2] (serological cases), p = 0.134. No correlation was found between the blood eosinophil count and serum PCR Ct values (Spearman r = -0.1341, p = 0.262).

**Table 4:** Sensitivity and agreement of serum PCR against microscopy and WB-based serology.

Reference method	*Schistosoma* serum PCR sensitivity (%)	Kappa coefficient	% Agreement	p^a^
Microscopy	32/34 (94.1%)	0.472	77.8	<0.0001
WB	71/98 (72.4%)	0.702	85.0	<0.0001

^a^p-value, Fisher’s exact test

The global agreement of serum PCR with Western Blot was higher than with microscopy (85%; Kappa = 0.702, Table 4). Interestingly, among WB-positive patients, the occurrence of a positive serum PCR was found to be correlated with ELISA and HA results (Table 5, p = 0.003). PCR was positive in only 1/5 sera with both negative ELISA and HA (20.0%), whereas serum PCR was positive in 17/18 (94.4%) sera with both positive tests. Lastly, 22/32 (68.8%) sera yielded positive PCR together with discordant ELISA and HA. ELISA indexes and HA titers were significantly higher when serum PCR was positive, with median values of 2.59 [1.91;3.29] *versus* 1.50 [0.91;1.90] (p<0.001), and 300 [0;320] *versus* 48 [0;160] (p = 0.04).

**Table 5:** Serum PCR performance according to combined ELISA and HA serological tests among patients with positive WB (N = 55^a^).

ELISA and HA results	PCR results n(%)		p^b^
	Negative	Positive	
Both negative tests (n = 5)	4 (80.0%)	1 (20.0%)	0.003
Discordant tests (n = 32)	10 (31.2%)	22 (68.8%)	
Both positive tests (n = 18)	1 (5.6%)	17 (94.4%)	

^a^HA and ELISA data were simultaneously available in 55/99 WB-positive sera.

^b^p-value, Chi-square test

Both Sm and ShPCRs were simultaneously positive in 6 serum samples (Table 6). For 2 patients (1 & 2) the presence of the two species was confirmed by microscopic examination of stools and urine. In patients 3 & 5 there was evidence of Sh infection in the bladder biopsy and urine, but stool analysis was not performed. In total, *S*. *mansoni* was unexpectedly detected in 3 serum specimens from patients with clinical symptoms evocative of urogenital schistosomiasis (patients 2, 3, and 5).

**Table 6:** Characteristics of patients with simultaneously positive Sm and ShPCRs in serum.

Patient No	Sex. age (yrs)	Visited endemic area	Length of stay (yrs)	Clinical symptoms	Clinical group	Ct values for serum PCR	Microscopy result	PCR in excreta
1	M. 55	South Africa. Mali	ND (traveler)	None	proven	Sh: 35.46	U: Sh	U: ND
						Sm: 34.70	S: Sm	S: Sm
2	M. 20	Guinea-Conakry	19	Hematuria	proven	Sh: 33.97	U: Sh+Si	U: ND
						Sm: >40	S: Sm	S: Sm
3	M. 18	Cameroon	12	Renal colic	proven	Sh: 36.26	Bladder biopsy: *Schistosoma sp*.	
						Sm: 37.04	U, S: ND	ND
4	M. 25	Sudan	24	None	probable	Sm: 36.12	U: ND	
						Sh: 33.57	S: -	ND
5	M. 15	Mali	15	Hematuria, micturition disorders	serological	Sh: 39.87	U: -	U: Sh
						Sm: 34.70	S: ND	S: ND
6	M. 15	Guinea	ND (migrant)	Abdominal pain, hematuria	serological	Sm: 32.13	U: -	U: -
						Sh: 30.64	S: -	S: ND

ND: not determined; U: urine; S: stools; -: negative; Sh: *S*. *haematobium*; Sm: *S*. *mansoni*; Si: *S*. *intercalatum*

### Comparison of PCR performances on serum and excreta

Among the 25 patients whose sera and urine were simultaneously screened for Sh, the positivity rate of PCR was slightly higher in serum compared to urine, 11/60 (18.3%) versus 8/60 (13.3%), (not statistically significant). For Sm detection, serum PCR yielded roughly similar results to stool PCR, detecting Sm in 21/48 (43.8%) and in 20/48 (41.7%), respectively (not statistically significant).

### Value of PCR for post-treatment follow-up

**Fig 2:**
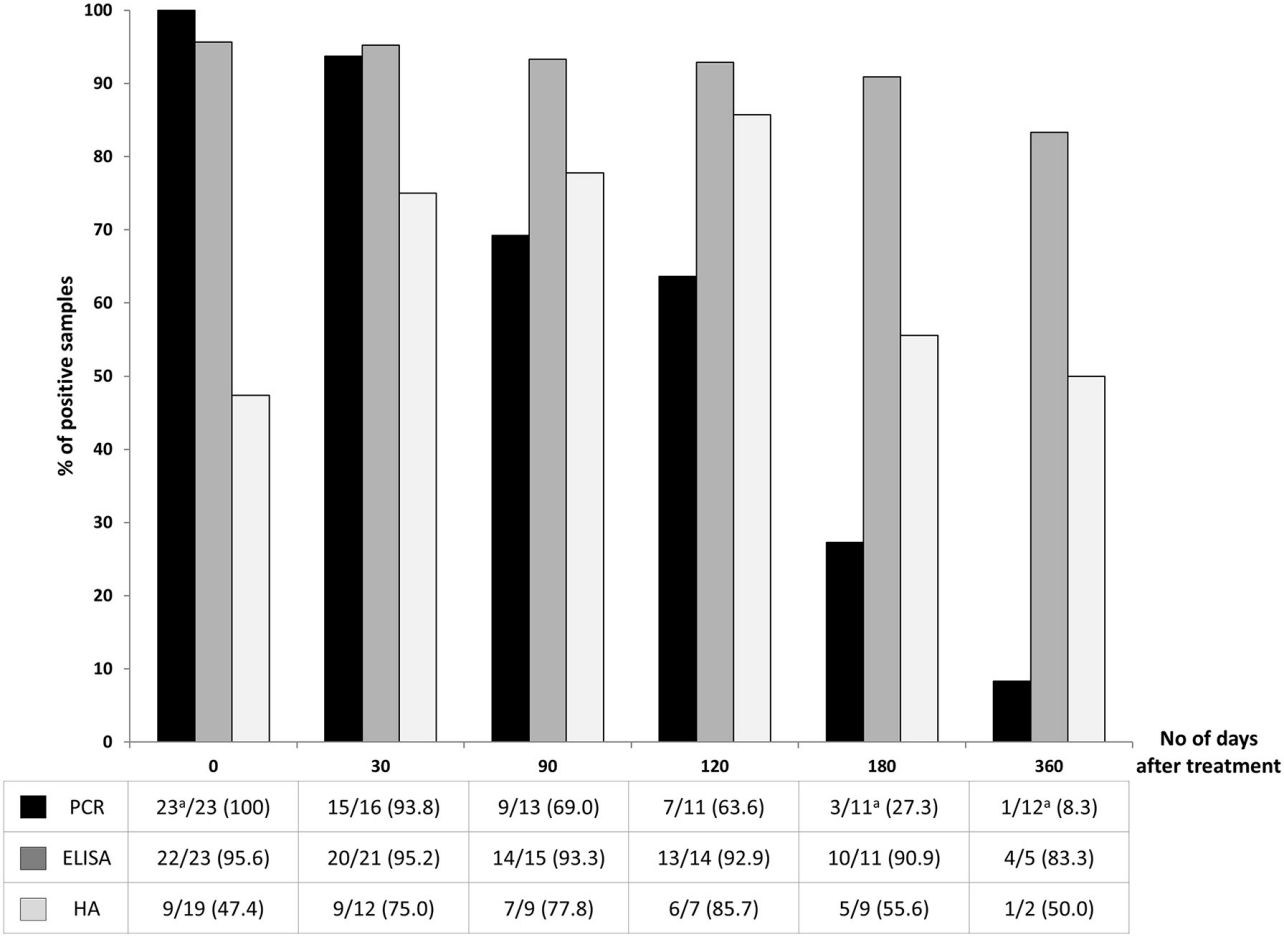
Kinetic of serum PCR, ELISA and HA positivity rates after treatment (n = 23 patients). Bars represent the percentage of positive samples which remained positive at different times post-treatment. The table presents the number of positive sera/number of analyzed sera, at each time (n/N (%)). ^a^One serum was positive for both Sm and ShPCR at diagnosis.

**Fig 3:**
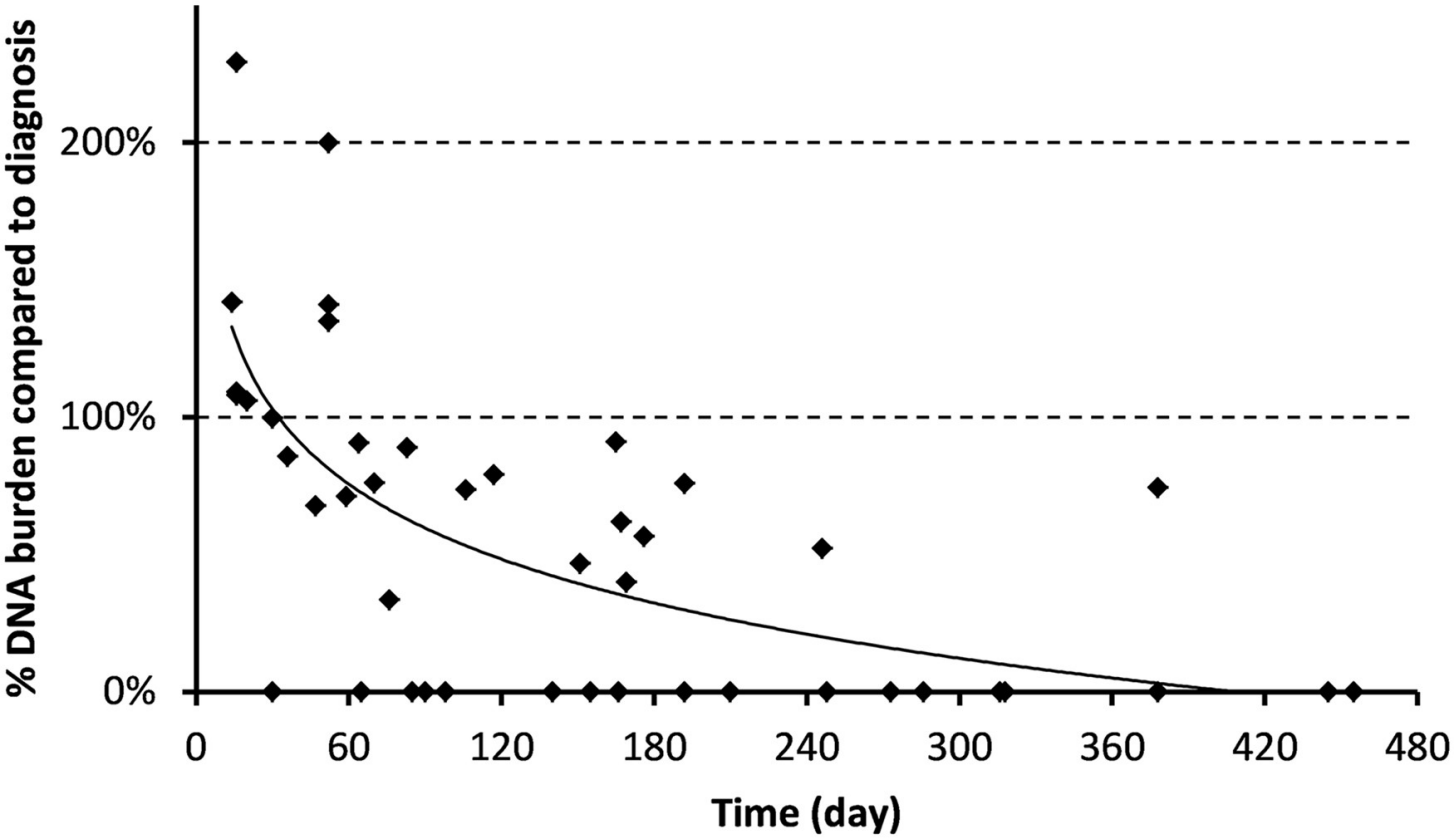
Relative value of serum DNA load after treatment compared to the initial load (day 0), according to the number of days post-treatment (n = 23 patients).

The last part of this study aimed at following the kinetics of a positive PCR on serum after treatment, to assess whether it could be a relevant tool for treatment monitoring, compared to serological techniques. Twenty-three patients benefited from follow-up serum samples (1 to 3) collected at different times after praziquantel therapy (14 to 455 days). One month after treatment, all but one serum remained positive by PCR (15/16, 93.8%), of which 9 were from proven cases (Fig 2). The patient who became negative as early as day 30 had a very low parasite burden before treatment (Ct-value for Sm PCR >40). The level of circulating *Schistosoma* DNA appeared to increase significantly within the first 60 days as shown in Fig 3. After 3, 4, and 6 months respectively, the positivity rate gradually declined to 69.0, 63.6 and 27.3% (Fig 2). One out of the 12 patients followed for more than 1 year after treatment (378 days) remained positive in serum.

By contrast, the positivity rate of serological tests ELISA and HA did not significantly decrease and remained constant within the first year after treatment. As shown in Fig 2, among the 96% of patients having a positive ELISA at day 0, up to 83% remained positive after 1 year. The HA positivity rate increased within the first four months after treatment, from 47% at day 0 to 86% at day 120, and remained at 50% after 1 year.

Such persistence of specific antibody detection, as well as DNA detection could not be linked with obvious treatment failure, since excreta remained positive with eggs for only 1/15 patients at day 30 and no further evidence of clinical relapse could be noticed.

Follow-up by PCR on stools was performed in two patients and did not detect persisting DNA two weeks after treatment, whereas PCR on serum was still positive at day 52 and day 83 post-treatment, respectively. By contrast, DNA burden in urine samples from 3 patients was strongly lowered but remained positive at day 60 (n = 3), and persisted until day 316 for one of them.

## Discussion

This study presents an evaluation of the performance of two *Schistosoma* PCR assays on excreta and serum samples, compared to microscopy and serology-based conventional methods for the diagnosis of imported intestinal and urogenital schistosomiasis.

Serum *Schistosoma* PCRs were previously shown to be mainly contributive for the diagnosis of early infections, as up to 90% of patients in acute invasion phase were positive, before detectable levels of eggs were excreted [26]. However, data on their clinical relevance are scarce, and the few existing reports referred to small patient cohorts. Hence, the strength of this work is to focus on a large cohort of chronically-infected patients and demonstrate the relevance of *Schistosoma* PCRs in sera as well as urine and stools.

Specificity of both PCR assays was firstly explored in excreta and sera and proved to be excellent (100%) in our cohort of subjects who never experienced any stay in known endemic areas. The analytical specificity of the ShPCR was previously demonstrated by Cnops *et al*., on a panel of 23 stool samples containing various intestinal parasites other than *Schistosoma* [25]. Similarly, the absence of cross-reaction with SmPCR was verified in a previous work [24]. Here, we confirmed the high specificity of both PCRs as none of the tested blood and intestinal parasites were cross-reactive with the schistosome tests.

In our study, PCRs assays greatly improved the detection of *Schistosoma* in urine and fecal samples, increasing the number of positive results by a factor of 2.6 and 1.4 for Sh and Sm respectively, compared to microscopy. This is consistent with previous studies which underlined the excellent performance of both *Dra* ShPCR in urine (PCR sensitivity of 36% *versus* 25% with microscopy on 401 samples [27]) and Sm1-7 PCR in stool samples (PCR sensitivity of 9.6% *versus* 0.9% by microscopy, on 572 samples [28]).

These two highly sensitive real-time PCRs have been the most used for clinical diagnosis, because of the huge number of gene copies in the *Schistosoma* genome. A few clinical evaluations have been reported on real-time PCRs targeting the ribosomal internal transcribed spacer 2 region (ITS 2), but these PCRs detect *Schistosoma sp*. without discrimination between *S*. *mansoni* and S. *haematobium*. This gene also displays interesting performances, as the PCR sensitivity was shown to range from 89–100% for Sh detection [18,29] and from 94–97% for Sm detection [30,31], compared to microscopy. The use of the 28S rDNA and mitochondrial regions such as *cox1* gene, have also been anecdotally described, but to a lesser extent [32,33].

All the specimens positive for *Schistosoma* eggs in urine or stools were confirmed by PCR assays. Conversely, among samples with an isolated positive PCR (negative microscopy), false-positivity of a PCR result was very unlikely, as all samples were from patients with epidemiological risk factors and positive WB-serology, consistent with *Schistosoma* infection. Additionally, the huge advantage of PCR on stools is to bypass the daily variation in egg excretion, which allows analyzing a single fecal sample, rather than 3 consecutive samples, as usually recommended. This also applies to urine samples, which can be collected from a unique micturition and not necessarily during a 24h-time period. Lastly, SmPCR was validated on biopsy samples with a concordance of 100% with microscopy, although it could only be done on a small number of specimens.

This work also presents a contribution of detection of circulating *Schistosoma* DNA to the current conventional diagnostic arsenal, based on serology and microscopic examination. A positive serum-based PCR is consistent with the parasite localization in blood vessels, the significant size of adult worms, and thus the expected high level of *Schistosoma* DNA in blood.

Here, in our cohort constituted mainly of African migrants, the PCR yield in serum exceeded 92% in patients with *Schistosoma*-positive urine or stools. Despite 2 false-negative results within the proven group, the global sensitivity of serum PCRs remained higher than that observed for microscopic techniques, as it was able to detect 77% and 49% of “probable active cases” and “strictly serological cases”, respectively.

The WB-based serology is a reference method for the serologic screening of schistosomiasis because of its sensitivity [8]. Surprisingly, when compared to serum PCR, WB yielded false-negative results in two patients, for whom the possibility of recent infection was excluded (African migrants who left endemic areas at least 4 months before). The first patient had a doubtful ELISA index, but a concomitant bladder biopsy containing living Sh eggs, and thus was a proven case. The other one had a positive serum Sm PCR together with a positive ELISA assay, but in the absence of excreta microscopy, the case was classified as excluded (Table 3), although true infection was likely.

Among WB-positive sera, the frequency of positive *Schistosoma* DNA detection in serum was found to be higher when the ELISA and HA tests were concomitantly positive. Among the serological approaches for the diagnosis of schistosomiasis, these two serological tests appear to yield more frequent positive antibody detections in case of active infection, compared to WB. Indeed, most former schistosomiasis patients with positive ELISA and HA assays are expected to turn negative [5,34] or to have a significantly lower antibody titer [35] within a variable period after an effective treatment. By contrast, LDBIO *Schistosoma* WB IgG is known to persist positive after treatment for a longer period than ELISA [36], probably for several years, as already observed with other parasitic WB assays based on IgG detection [37,38].

The great advantage of serum PCR is to provide species identification even when microscopic examination is negative, allowing targeted clinical work-up to identify complications of long- term infections.

Direct species identification by serum PCR allowed diagnosis of mixed infection in 6 patients. Positive results by the 2 PCR assays were unlikely to be due to cross-reaction between the 2 species, as previous studies demonstrated the species-specificity of these assays [24,25]. Two hypotheses can be considered to explain this dual positivity. First, we can assume that these patients, all coming from Africa, were simultaneously infected with Sh and Sm, as frequently observed in several endemic African countries, with up to 50% in a Senegalese study from Meurs *et al*. [39]. Unfortunately, this hypothesis could be confirmed for two patients only, for whom both Sm and Sh eggs were detected in excreta. For the 4 remaining patients, stool and urine samples were not available for simultaneous screening.

Secondly, the positivity of both targets in serum could also be related to an infection involving a hybrid between *S*. *mansoni* and *S*. *haematobium*. Besides, two urine samples from the same patient also yielded both positive Sm and Sh PCRs, at low Ct values (17 and 18 for Sh and SmPCR, respectively), whereas only Sh eggs were detected by microscopy. The detection of DNA from both species in urine suggested a Sm-Sh hybrid infection in this African patient. Moreover, recent works also demonstrated the emergence of such schistosome hybrids in regions where the parasite burden is high, either between cattle and human schistosomes [4,40], or between *S*. *mansoni* and *S*. *haematobium* [41,42]. Thus, the impact of hybrid species on performances of diagnostic tools should be addressed, to determine whether they could generate cross-reaction with both PCRs assays. *Schistosoma* eggs from one of the 3 patients with a previous stay in Corsica were genotyped as *S*. *haematobium-S*. *bovis* hybrids [4,43]. For 2 of the patients coming from Corsica, including the one with the hybrid, ShPCR was positive in urine and/or serum, demonstrating the performance of this PCR for hybrid species detection.

Apart from assessing the diagnostic performances of serum PCRs, this study also evaluated their relevance for follow-up after treatment, and ability to discriminate between ongoing or previous infections, that cannot be performed with WB-based assays. In this study, the analysis of sequential sera from praziquantel-treated patients strikingly demonstrated the potentially long persistence of *Schistosoma* circulating DNA without any link with clinical or biological failure. Indeed, 27% remained positive at 6 months, and 8% one year after an adequate cure. In addition, none of these patients were treated during the invasion stage, during which schistosomula are known to be less susceptible to praziquantel [44,45], and which could have explained a therapeutic failure.

Several hypotheses could explain why *Schistosoma* DNA is still detected a long time after treatment. Firstly, the parasite DNA could continue to be released from slowly degenerating eggs that are trapped in tissues, or from killed worms. Therefore, after treatment, no eggs are detected or at least no living eggs, but parasite DNA is not totally cleared and might remain detectable by high sensitive *Schistosoma* real-time PCR assays. Secondly, the incomplete cure may result from the sub-curative effect of praziquantel when used at usual doses [46]. Finally, a recent review from Lu *et al*. [47] raises the possibility of single-sex schistosome infection that could partly explain why antigen and/or DNA detection could occur without egg detection. Indeed, after a single-sex cercarial exposure, male or female worms will be able to persist within the host without producing eggs, and thus cause asymptomatic infection. While reports on the emergence of low sensitive *Schistosoma* strains to praziquantel are still scarce [48], single-sex schistosomes, especially females, have been demonstrated to be largely refractory to praziquantel compared to paired ones, in both *in vitro* and *in vivo* murine models [44]. That’s why, despite an appropriate praziquantel treatment, the persistence of single-sex schistosomes less susceptible to this anti-parasitic drug, could still generate a positive PCR, without any egg-laying or clinical symptoms.

Other clinical studies reported long-lasting PCR positivity after treatment in a large mixed cohort studied by Wichmann *et al* [24], and in an urogenital case, until more than 300 days after the first praziquantel treatment [49]. These studies also emphasized the contrasting results of persistent DNA detection, compared to antigen detection which has been shown to become negative in serum or urine within a few days or weeks after treatment [50,51]. Cnops *et al*. also reported that ShPCR remained positive for at least 3 months (n = 8 patients) with a decreasing signal after treatment (higher Ct-values), and they pointed out PCR’s potential to monitor treatment [25].

Here, an evaluation of *Schistosoma* serum PCR to monitor treatment has been made by comparison to serological assays. During a follow-up period limited to 1 year, the serum PCR slowly turned negative in most patients, whereas ELISA and HA-based antibody detection remained above the positivity cut-off. These results suggest that at early times post-treatment (within the first months), there is no relevant serum biomarker for follow-up and excreta analysis remains essential. Nevertheless, more than one year after treatment, serum PCR could possibly be an indicator of effective treatment, better than conventional serological tests.

In conclusion, our work emphasized the high performance of targeted *Schistosoma* PCRs in excreta, greatly improving the diagnosis of both urogenital and intestinal schistosomiasis. Besides, combining serum PCR with conventional serological assays contributes added value to confirming diagnosis and providing species identification to guide further clinical exploration. Finally, *Schistosoma* serum PCR could also be useful to monitor the outcome when performed much later than an anti-*Schistosoma* treatment (about one year), as a negative serum PCR may demonstrate a cured infection.

## Supporting information

S1 STARD Checklist (DOCX)

## References

[1] Colley DG, Bustinduy AL, Secor WE, King CH. Human schistosomiasis. The Lancet. 2014;383:2253–64. 10.1016/S0140-6736(13)61949-2 PMC4672382 24698483

[2] Ross AG, Chau TN, Inobaya MT, Olveda RM, Li Y, Harn D. A new global strategy for the elimination of schistosomiasis. International Journal of Infectious Diseases. 2017;54:130–7. 10.1016/j.ijid.2016.09.023 27939558

[3] Berry A, Moné H, Iriart X, Mouahid G, Aboo O, Boissier J, et al *Schistosomiasis haematobium*, Corsica, France. Emerg Infect Dis. 2014;20(9):1595–7. 10.3201/eid2009.140928 25153697 PMC4178424

[4] Boissier J, Grech-Angelini S, Webster BL, Allienne J-F, Huyse T, Mas-Coma S, et al Outbreak of urogenital schistosomiasis in Corsica (France): an epidemiological case study. The Lancet Infectious Diseases. 2016;16(8):971–9. 10.1016/S1473-3099(16)00175-4 27197551

[5] Hinz R, Schwarz NG, Hahn A, Frickmann H. Serological approaches for the diagnosis of schistosomiasis–A review. Molecular and Cellular Probes. 2017;31:2–21. 10.1016/j.mcp.2016.12.003 27986555

[6] Agbata EN, Morton RL, Bisoffi Z, Bottieau E, Greenaway C, Biggs B-A, et al Effectiveness of Screening and Treatment Approaches for Schistosomiasis and Strongyloidiasis in Newly-Arrived Migrants from Endemic Countries in the EU/EEA: A Systematic Review. Int J Environ Res Public Health. 2018;16(1). 10.3390/ijerph16010011 PMC6339107 30577567

[7] Berry A, Fillaux J, Martin-Blondel G, Boissier J, Iriart X, Marchou B, et al Evidence for a permanent presence of schistosomiasis in Corsica, France, 2015. Eurosurveillance. 2016;21(1). 10.2807/1560-7917.ES.2016.21.1.30100 26767427

[8] Beltrame A, Guerriero M, Angheben A, Gobbi F, Requena-Mendez A, Zammarchi L, et al Accuracy of parasitological and immunological tests for the screening of human schistosomiasis in immigrants and refugees from African countries: An approach with Latent Class Analysis. PLOS Neglected Tropical Diseases. 2017;11(6):e0005593 10.1371/journal.pntd.0005593 28582412 PMC5472324

[9] Fuss A, Mazigo HD, Tappe D, Kasang C, Mueller A. Comparison of sensitivity and specificity of three diagnostic tests to detect *Schistosoma mansoni* infections in school children in Mwanza region, Tanzania. PlosOne. 2018;13(8):14. 10.1371/journal.pone.0202499 PMC6105001 30133490

[10] Utzinger J, Becker SL, van Lieshout L, van Dam GJ, Knopp S. New diagnostic tools in schistosomiasis. Clinical Microbiology and Infection. 2015;21(6):529–42. 10.1016/j.cmi.2015.03.014 25843503

[11] Ogongo P, Kariuki TM, Wilson RA. Diagnosis of schistosomiasis *mansoni*: an evaluation of existing methods and research towards single worm pair detection. Parasitology. 2018;1–12. 10.1017/S0031182018000240 29506583

[12] Rubaba O, Chimbari MJ, Soko W, Manyangadze T, Mukaratirwa S. Validation of a urine circulating cathodic antigen cassette test for detection of *Schistosoma haematobium* in uMkhanyakude district of South Africa. Acta Tropica. 2018;182:161–5. 29486172 10.1016/j.actatropica.2018.02.029

[13] He P, Song L, Xie H, Liang J, Yuan D, Wu Z, et al Nucleic acid detection in the diagnosis and prevention of schistosomiasis. Infectious Diseases of Poverty. 2016;5(1). 10.1186/s40249-016-0116-y PMC4812660 27025210

[14] Cavalcanti MG, Cunha AFA, Peralta JM. The Advances in Molecular and New Point-of-Care (POC) Diagnosis of Schistosomiasis Pre- and Post-praziquantel Use: In the Pursuit of More Reliable Approaches for Low Endemic and Non-endemic Areas. Front Immunol. 2019;10:858 10.3389/fimmu.2019.00858 31191512 PMC6546849

[15] Pontes LA, Oliveira MC, Katz N, Dias-Neto E, Rabello ANA. Comparison of a polymerase chain reaction and the Kato-Katz technique for diagnosing infection with *Schistosoma mansoni*. The American journal of tropical medicine and hygiene. 2003;68(6):652–656. 12887022

[16] Oliveira LMA, Santos HLC, Gonçalves MML, Barreto MGM, Peralta JM. Evaluation of polymerase chain reaction as an additional tool for the diagnosis of low-intensity *Schistosoma mansoni* infection. Diagnostic Microbiology and Infectious Disease. 2010;68(4):416–21. 10.1016/j.diagmicrobio.2010.07.016 20884153

[17] de Carvalho GC, dos Marques LHS, Gomes LI, Rabello A, Ribeiro LC, Scopel KKG, et al Polymerase chain reaction for the evaluation of *Schistosoma mansoni* infection in two low endemicity areas of Minas Gerais, Brazil. Memórias do Instituto Oswaldo Cruz. 2012;107(7):899–902. 10.1590/s0074-02762012000700010 23147146

[18] Obeng BB, Aryeetey YA, de Dood CJ, Amoah AS, Larbi IA, Deelder AM, et al Application of a circulating-cathodic-antigen (CCA) strip test and real-time PCR, in comparison with microscopy, for the detection of *Schistosoma haematobium* in urine samples from Ghana. Ann Trop Med Parasitol. 2008;102(7):625–33. 10.1179/136485908X337490 18817603

[19] Hamburger J, Turetski T, Kapeller I, Deresiewicz R. Highly repeated short DNA sequences in the genome of *Schistosoma mansoni* recognized by a species-specific probe. Molecular and Biochemical Parasitology. 1991;44(1):73–80. 10.1016/0166-6851(91)90222-r 2011155

[20] Hamburger J, He-Na, Abbasi I, Ramzy RM, Jourdane J, Ruppel A. Polymerase chain reaction assay based on a highly repeated sequence of *Schistosoma haematobium*: a potential tool for monitoring schistosome-infested water. Am J Trop Med Hyg. 2001;65(6):907–11. 10.4269/ajtmh.2001.65.907 11791997

[21] Code de la Santé Publique. Décret n° 2017–884 du 9 mai 2017 modifiant certaines dispositions réglementaires relatives aux recherches impliquant la personne humaine | Legifrance. Article R.1121-1-1. 2017;https://www.legifrance.gouv.fr/eli/decret/2017/5/9/AFSP1706303D/jo/texte.

[22] ANOFEL, Houzé S, Botterel-Chartier F. Parasitologie et mycologie médicales—Guide des analyses et des pratiques diagnostiques. Elsevier Health Sciences. 2018.

[23] Fabre R, Berry A, Morassin B, Magnaval JF. Comparative assessment of conventional PCR with multiplex real-time PCR using SYBR Green I detection for the molecular diagnosis of imported malaria. Parasitology. 2004;128(Pt 1):15–21. 10.1017/s0031182003004219 15002899

[24] Wichmann D, Panning M, Quack T, Kramme S, Burchard G-D, Grevelding C, et al Diagnosing Schistosomiasis by Detection of Cell-Free Parasite DNA in Human Plasma. PLoS Neglected Tropical Diseases. 2009;3(4):e422 10.1371/journal.pntd.0000422 19381285 PMC2667260

[25] Cnops L, Soentjens P, Clerinx J, Van Esbroeck M. A *Schistosoma haematobium*-Specific Real-Time PCR for Diagnosis of Urogenital Schistosomiasis in Serum Samples of International Travelers and Migrants. PLoS Neglected Tropical Diseases. 2013;7(8):e2413 10.1371/journal.pntd.0002413 24009791 PMC3757062

[26] Wichmann D, Poppert S, Thien HV, Clerinx J, Dieckmann S, Jensenius M, et al Prospective European-wide multicentre study on a blood based real-time PCR for the diagnosis of acute schistosomiasis. BMC Infectious Diseases. 2013;13(1):55. 23363565 10.1186/1471-2334-13-55 PMC3563621

[27] Ibironke O, Koukounari A, Asaolu S, Moustaki I, Shiff C. Validation of a New Test for *Schistosoma haematobium* Based on Detection of *Dra1* DNA Fragments in Urine: Evaluation through Latent Class Analysis. PLoS Neglected Tropical Diseases. 2012;6(1):e1464 10.1371/journal.pntd.0001464 22235360 PMC3250497

[28] Espírito-Santo MCC, Alvarado-Mora MV, Dias-Neto E, Botelho-Lima LS, Moreira JP, Amorim M, et al Evaluation of real-time PCR assay to detect *Schistosoma mansoni* infections in a low endemic setting. BMC Infectious Diseases. 2014;14(1). 10.1186/s12879-014-0558-4 PMC4210485 25338651

[29] Vinkeles Melchers NVS, van Dam GJ, Shaproski D, Kahama AI, Brienen EAT, Vennervald BJ, et al Diagnostic Performance of Schistosoma Real-Time PCR in Urine Samples from Kenyan Children Infected with *Schistosoma haematobium*: Day-to-day Variation and Follow-up after Praziquantel Treatment. PLoS Neglected Tropical Diseases. 2014;8(4):e2807 10.1371/journal.pntd.0002807 24743389 PMC3990496

[30] Schunk M, Kebede Mekonnen S, Wondafrash B, Mengele C, Fleischmann E, Herbinger K-H, et al Use of Occult Blood Detection Cards for Real-Time PCR-Based Diagnosis of Schistosoma Mansoni Infection. PLoS ONE. 2015;10(9):e0137730 10.1371/journal.pone.0137730 26360049 PMC4567332

[31] Meurs L, Polderman AM, Vinkeles Melchers NVS, Brienen EAT, Verweij JJ, Groosjohan B, et al Diagnosing Polyparasitism in a High-Prevalence Setting in Beira, Mozambique: Detection of Intestinal Parasites in Fecal Samples by Microscopy and Real-Time PCR. PLoS Negl Trop Dis. 2017;11(1):e0005310 10.1371/journal.pntd.0005310 28114314 PMC5289637

[32] Cnops L, Tannich E, Polman K, Clerinx J, Van Esbroeck M. *Schistosoma* real-time PCR as diagnostic tool for international travellers and migrants. Tropical Medicine & International Health. 2012;17(10):1208–16. 22882536 10.1111/j.1365-3156.2012.03060.x

[33] ten Hove RJ, Verweij JJ, Vereecken K, Polman K, Dieye L, van Lieshout L. Multiplex real-time PCR for the detection and quantification of *Schistosoma mansoni* and *S*. *haematobium* infection in stool samples collected in northern Senegal. Trans R Soc Trop Med Hyg. 2008;102(2):179–85. 10.1016/j.trstmh.2007.10.011 18177680

[34] Zhu Y-C. Immunodiagnosis and its role in schistosomiasis control in China: a review. Acta Tropica. 2005;96(2–3):130–6. 10.1016/j.actatropica.2005.07.007 16143288

[35] Mott KE, Dixon H. Collaborative study on antigens for immunodiagnosis of schistosomiasis. Bull World Health Organ. 1982;60(5):729–53. 6983926 PMC2536043

[36] LDBioDiagnostics. Schistosoma Western Blot IgG, LDBIO. Instructions for use. [Internet]. 2014. https://ansm.sante.fr/var/ansm_site/storage/original/application/cfda51c46f3b1bb41471ec9b7d9dc6ec.pdf

[37] Kumar Tenguria R, Naik MI, Fomda B. Application of Western Blotting for the Post-Treatment Monitoring of Human Cystic Echinococcosis. Iran J Public Health. 2013;42(8):826–32. 26056636 PMC4441913

[38] Rubinsky-Elefant G, Hoshino-Shimizu S, Jacob CMA, Sanchez MCA, Ferreira AW. Potential immunological markers for diagnosis and therapeutic assessment of toxocariasis. Rev Inst Med Trop Sao Paulo. 2011;53(2):61–5. 10.1590/s0036-46652011000200001 21537750

[39] Meurs L, Mbow M, Vereecken K, Menten J, Mboup S, Polman K. Epidemiology of mixed *Schistosoma mansoni* and *Schistosoma haematobium* infections in northern Senegal. Int J Parasitol. 2012;42(3):305–11. 10.1016/j.ijpara.2012.02.002 22366733

[40] Boon NAM, VAN DEN Broeck F, Faye D, Volckaert FAM, Mboup S, Polman K, et al Barcoding hybrids: heterogeneous distribution of *Schistosoma haematobium* × *Schistosoma bovis* hybrids across the Senegal River Basin. Parasitology. 2018;145(5):634–45. 10.1017/S0031182018000525 29667570

[41] Huyse T, Van den Broeck F, Hellemans B, Volckaert FAM, Polman K. Hybridisation between the two major African schistosome species of humans. International Journal for Parasitology. 2013;43(8):687–9. 10.1016/j.ijpara.2013.04.001 23643461

[42] Le Govic Y, Kincaid-Smith J, Allienne J-F, Rey O, de Gentile L, Boissier J. *Schistosoma haematobium-Schistosoma mansoni* Hybrid Parasite in Migrant Boy, France, 2017. Emerging Infect Dis. 2019;25(2):365–7. 10.3201/eid2502.172028 30526763 PMC6346478

[43] Ramalli L, Mulero S, Noël H, Chiappini J-D, Vincent J, Barré-Cardi H, et al Persistence of schistosomal transmission linked to the Cavu river in southern Corsica since 2013. Euro Surveill. 2018;23(4). 10.2807/1560-7917.ES.2018.23.4.18-00017 PMC5801336 29382413

[44] Pica-Mattoccia L, Cioli D. Sex- and stage-related sensitivity of *Schistosoma mansoni* to *in vivo* and *in vitro* praziquantel treatment. Int J Parasitol. 2004;34(4):527–33. 10.1016/j.ijpara.2003.12.003 15013742

[45] Aragon AD, Imani RA, Blackburn VR, Cupit PM, Melman SD, Goronga T, et al Towards an understanding of the mechanism of action of praziquantel. Mol Biochem Parasitol. 2009;164(1):57–65. 10.1016/j.molbiopara.2008.11.007 19100294 PMC2886009

[46] Doenhoff MJ. Is Schistosomicidal Chemotherapy Sub-curative? Implications for Drug Resistance. Parasitol Today. 1998;14(10):434–5. 17040836 10.1016/s0169-4758(98)01315-5

[47] Lu D-B, Deng Y, Ding H, Liang Y-S, Webster JP. Single-sex schistosome infections of definitive hosts: Implications for epidemiology and disease control in a changing world. PLOS Pathogens. 2018;14(3):e1006817 10.1371/journal.ppat.1006817 29494686 PMC5833269

[48] Melman SD, Steinauer ML, Cunningham C, Kubatko LS, Mwangi IN, Wynn NB, et al Reduced Susceptibility to Praziquantel among Naturally Occurring Kenyan Isolates of *Schistosoma mansoni*. PLoS Neglected Tropical Diseases. 2009;3(8):e504 10.1371/journal.pntd.0000504 19688043 PMC2721635

[49] Kato-Hayashi N, Yasuda M, Yuasa J, Isaka S, Haruki K, Ohmae H, et al Use of Cell-Free Circulating Schistosome DNA in Serum, Urine, Semen, and Saliva To Monitor a Case of Refractory Imported Schistosomiasis Hematobia. J Clin Microbiol. 2013;51(10):3435–8. 10.1128/JCM.01219-13 23884992 PMC3811636

[50] De Clercq D, Sacko M, Vercruysse J, vanden Bussche V, Landouré A, Diarra A, et al Assessment of cure by detection of circulating antigens in serum and urine, following schistosomiasis mass treatment in two villages of the Office du Niger, Mali. Acta Tropica. 1997;68(3):339–46. 9492918 10.1016/s0001-706x(97)00111-3

[51] Kildemoes AO, Vennervald BJ, Tukahebwa EM, Kabatereine NB, Magnussen P, de Dood CJ, et al Rapid clearance of *Schistosoma mansoni* circulating cathodic antigen after treatment shown by urine strip tests in a Ugandan fishing community–Relevance for monitoring treatment efficacy and re-infection. PLOS Neglected Tropical Diseases. 2017;11(11):e0006054 10.1371/journal.pntd.0006054 29131820 PMC5703575

